# The acceptability, adherence, and preliminary effectiveness of a digital treatment for adolescents with subthreshold eating disorders. Findings from an open feasibility trial in routine clinical care

**DOI:** 10.1186/s40337-026-01596-9

**Published:** 2026-04-09

**Authors:** Guri Holgersen, Emilie S. Nordby, Ester Marie S. Espeset, Tine Nordgreen

**Affiliations:** 1 Division of Psychiatry, Haukeland University Hospital, Bergen, Norway; 2 Department of Global Public Health and Primary Care, University of Bergen, Bergen, Norway; 3 Department of Child and Adolescent Psychiatry, Helse Fonna, Haugesund, Norway

**Keywords:** Eating disorders, Adolescents, Transdiagnostic, Digital treatment, Routine clinical care, Developing novel intervention, Feasibility, Acceptability, Adherence, Preliminary effectiveness

## Abstract

**Background:**

Digital interventions have the potential to enhance treatment for eating disorders. Yet research remains limited, especially among adolescents. The aim of the present study was to evaluate whether a therapist-guided digital treatment for adolescents with subthreshold eating disorders was feasible within routine clinical care. The primary objectives of the trial were to evaluate the acceptability, adherence, and preliminary effectiveness of the novel intervention.

**Methods:**

An open feasibility trial of a novel digital treatment was conducted within routine clinical care. Participants were adolescents aged 15–18 years with subthreshold eating disorders. Acceptability was assessed among the adolescents during and post treatment, adherence was evaluated through engagement with treatment modules and dropout, whereas preliminary effectiveness was assessed by examining positive and negative symptoms change from pre to post treatment. Primary clinical outcomes included eating disorder symptomatology and psychosocial impairment, whereas secondary outcomes were quality of life, emotion dysregulation, depression, and anxiety.

**Results:**

A total of 24 adolescent girls participated in the digital treatment. The mean age was 16 years (range: 15–18, SD: 0.78). The findings suggest that the treatment was feasible, with favourable acceptability ratings and satisfactory adherence. The linear mixed model analyses showed significant improvement in eating disorder symptomatology and social impairment. Non-significant improvements were observed for the secondary clinical outcomes. Importantly, no serious negative effects were reported.

**Conclusions:**

The findings suggest that the therapist-guided digital treatment for adolescents with subthreshold eating disorders is both acceptable and well tolerated within routine clinical care, underscoring the potential of digital approaches to effectively complement existing treatment care.

*Trial registration*: ClinicalTrials.gov: NCT06306586. Approved: 05.03.2024. Available online: 12.03.2024.

**Supplementary Information:**

The online version contains supplementary material available at 10.1186/s40337-026-01596-9.

## Abstract (plain language summary)

This study looked at whether a new digital treatment for eating disorders could work well for adolescents in regular healthcare settings. The treatment was guided by a therapist and offered through a mobile application. Twenty-four girls between the ages of 15 and 18 took part in the intervention. Most of the adolescents reported to be satisfied with the treatment and found it helpful. The majority remained engaged with the program and completed most of the sessions. The results showed that their eating disorder symptoms and the impact these symptoms had on their daily lives improved during the treatment. Importantly, no serious negative effects were reported. This suggests that digital treatments can be a safe and useful option for adolescents with eating disorders and may work well alongside traditional care.

**Supplementary Information:**

The online version contains supplementary material available at 10.1186/s40337-026-01596-9.

## Background

Eating disorders often emerge during adolescence and are associated with profound negative psychological and physical consequences [1, 2]. The disorders hinder cognitive, emotional, and social development, and are associated with reduced quality of life and an increased risk of suicidality [1, 3–5]. Eating disorders also have one of the highest rates of medical complications compared to other psychiatric illnesses and are often accompanied by severe comorbid mental health disorders [1, 2]. Early intervention is crucial in treating eating disorders, and several evidence-based treatments are available for children and adolescents [1, 6]. However, numerous individual and systemic barriers hinder access to care, and current treatment approaches are characterised by high rates of drop-out and low rates of remission [7–9].

Treatments delivered via computers or smartphones, often referred to as digital interventions or digital treatments, have potential to enhance the accessibility and efficiency of healthcare systems [10, 11]. Digital interventions have demonstrated effectiveness across a wide range of mental health conditions [12–14], yielding outcomes comparable to those of traditional face-to-face treatments [12–14]. Promising results also exist for digital treatments for eating disorders [15–17]. In addition to demonstrate significant effects in eating disorder symptoms [15, 18], they offer a more accessible model of delivering psychological care by extending treatment beyond in-person sessions [10, 15] and can reduce barriers to treatment-seeking [1, 19].

There is growing interest in the use of smartphone applications for eating disorder treatment [20]. App-based interventions may be delivered as an adjunct to face-to-face therapy or as standalone interventions with or without therapist guidance [19]. In standalone therapist guided interventions, clinicians provide synchronous (e.g., telephone or video) or asynchronous (e.g., messaging or email) support through the program. The digital intervention is the primary therapeutic component, with the therapist acting as a support or facilitator to enhance engagement, adherence, and effective use rather than delivering full psychotherapy. In standalone self-guided interventions, the participants complete independently. All therapeutic content, exercises, and guidance are built into the program itself, and there is no contact with a therapist or health professional. App-based interventions allow individuals to engage with treatment at their own pace, which may enhance their sense of autonomy and control over the therapeutic process [20], factors known to improve adherence to therapeutic interventions [20, 21]. Delivering therapy via smartphone applications may be particularly appealing to adolescents with eating disorders, given their familiarity with and frequent use of digital technologies. In Norway, 97% of adolescents aged 9 to 18 have their own mobile phone [22] and 79% have used the internet to search for health-related information [23].

Despite app-based interventions potential in eating disorder treatments, rigorous evaluations of their efficacy or effectiveness among clinical samples remain limited [20, 24]. Among adolescents only two studies have tested the effectiveness of app-based interventions [25, 26]. Anastasiadou et al. compared an app-based intervention (TCApp) in adjunction with standard face-to-face CBT to treatment as usual in a transdiagnostic sample [25]. The multicentred RCT found no significant differences between the two groups in eating disorder symptomatology and reported that CBT was associated with reduced symptom severity regardless of treatment condition [25]. In the other study Neumayr et al. (2019) evaluated a therapist-guided aftercare intervention for inpatients with Anorexia Nervosa [26]. The app-based intervention was Recovery Record, a widely used application for tracking food intake and other eating disorder related behaviours [27]. The pilot RCT compared the app-based intervention in adjunction to treatment as usual to treatment as usual alone [26]. The study demonstrated high adherence and acceptance of the application and the aftercare intervention [26]. However, consistent with findings from the TCApp study, the use of Recovery Record as an adjunct to standard therapy did not yield additional benefits beyond those achieved with therapy alone [26]. The current evidence does not adequately address the efficacy of apps-based intervention for adolescents with eating disorders [16, 28]. To maximize the clinical utility of these interventions more clinical trials are warranted [16, 28].

To address the gaps in existing care and harness the potential of digital treatments, it is essential to develop novel user-friendly interventions [17, 21]. Even more important is ensuring that their potential is realised in a real-world setting [20, 29]. Evaluating the feasibility of novel interventions in routine clinical care is therefore a crucial step in determining whether it can be implemented in the health services [29, 30]. In this study, the aim was to evaluate whether a novel therapist-guided digital treatment for adolescents with subthreshold eating disorders was feasible within routine clinical care. The primary objectives of the trial were to evaluate the acceptability, adherence, and preliminary effectiveness of the novel intervention.

## Methods

### Study design

This was an open feasibility trial conducted in routine clinical care. The study was conducted in line with the UK Medical Research Council’s guidance for developing and evaluating complex interventions and adheres to the CONSORT guidelines for feasibility trials [see, Additional file 1 for checklist] [29, 31].

### Study setting

The study was conducted within the Western Norway Regional Health Authority (Helse Vest RHF), which is part of the state-funded and governed Norwegian specialist health care system. Participants were patients at Child and Adolescent Mental Health Services (CAMHS) at Haukeland University Hospital and Helse Fonna. These services serve both urban and rural areas across 26 municipalities, with a population of approximate 122 000 youth below 18 years [32]. Participants were recruited between March 2024 and May 2025, and final post-treatment assessment data were collected in August 2025.

### Sample

In line with the feasibility trial design, no formal sample size calculation was conducted. However, this study aimed to recruit approximate 30 participants, as this number was assumed sufficient to assess the feasibility of the novel intervention, based on sample sizes used in previous studies of digital interventions for eating disorders [33, 34]. Inclusion and exclusion criteria are described in Table 1. Although the digital treatment was designed for adolescents with eating disorders, the present study included only participants with subthreshold eating disorders. This study was the first clinical trial to evaluate a digital treatment for adolescents with eating disorders within routine clinical care in Norway. During the planning phase, there were concerns about enrolling participants who were not medically stable, given the potential risks associated with untreated medical complications in this population. A more flexible inclusion strategy, allowing clinicians to judge suitability for a digital intervention, was initially proposed but could not be implemented due to the assumption that this would threat the study’s internal validity. To address these concerns, the study excluded adolescents with anorexia nervosa and bulimia nervosa. Avoidant restrictive food intake disorder (AFRID) was an exclusion criterion due to the clinical picture difference from the other eating disorders [35]. Participants were withdrawn from the study if they met any of the exclusion criteria at any point during the treatment.

**Table 1:** Inclusion and exclusion criteria

Inclusion criteria	Exclusion criteria
Age 15–18 years	Anorexia nervosa
Atypical anorexia nervosa	Bulimia nervosa
Atypical bulimia nervosa	Avoidant restrictive food intake disorders
Binge eating disorder	Receiving inpatient treatment
Eating disorder, unspecified	Receiving face-to-face psychological treatment
Stable dose of medication for a co-morbid psychiatric disorder for six weeks	Co-morbid medical condition or disorder known to influence eating or weight (i.e., pregnancy, cancer)
Mobile phone with internet access	Acute suicidality and severe depressive episode
Speaks and writes Norwegian	Substance abuse and substance dependence
	Psychotic disorder

### Procedure

Information about the study was distributed at eight child and adolescence psychiatric outpatient clinics within the study’s catchment area. Interested adolescents accessed a brief preliminary online screening via a QR code. The online screening assessed disturbance in eating, eating-related behaviours, distress in daily life and three of the inclusion criteria (age, internet access, and language). Eligible participants were contacted by telephone for further assessment of inclusion and exclusion criteria using the Eating Disorder Assessment for DSM-5 (EDA-5) [36] and The Mini international neuropsychiatric interview (MINI) [37]. Due to slow recruitment, the study also informed about the study at upper secondary schools and through social media. All participants were required to be eligible for specialised care in accordance with national priority guidelines [38], thus participants who were not already patients at CAMHS were required to visit their general practitioner (GP) to obtain a referral to the study. Eligible participants were granted access to the intervention where they signed the consent form, completed pre-treatment measures, and were introduced to the intervention through an initial information module. Participants aged 15 years old was required to provide additional parental consent. The intervention was delivered as a native application compatible with both iOS and Android operating systems that could be installed directly onto a mobile device. Within 14 days following completion of the pre-treatment measures, the participants had their first video consultation with their therapist.

### Intervention

The novel intervention being studied in this clinical trial was eBalance, a therapist-guided digital treatment delivered via a smartphone application. eBalance was developed based on the perspectives of adolescents with lived experience of eating disorders, feedback from clinicians and the existing evidence base on eating disorders, psychological treatments and digital interventions [39–41]. It adopts a transdiagnostic cognitive-behavioural maintenance model [42] with components of emotion regulation approaches also included. The treatment started with an initial clinical assessment session conducted via video consultation. The purpose of the session was to establish a therapeutic alliance, introduce the adolescent to eBalance and conduct a clinical assessment. Provided that both the clinician and the adolescent considered the treatment to be appropriate, the adolescent was given access to the treatment. Adolescents progressed sequentially through eight modules, receiving weekly clinician support via scheduled 15-to-30-min telephone consultations. The decision to incorporate synchronous therapist contact (via telephone and video) was informed by input from adolescents with lived experience of eating disorders as well as feedback from clinicians. Modules were unlocked on a weekly basis, except for modules three and five, which were designed to be completed over a two-week period. An overview of the intervention content and proposed mechanisms of change are presented in Table 2.

**Table 2:** Overview of the intervention

Modules	Brief description	Proposed mechanisms
Information module	The treatments development and overarching aim. The application’s functionalities. Guidance on use	Increasing insight and motivation
1. What is an eating disorder?	The nature and consequences of eating disorders. Identifying disordered eating patterns. Developing a coping plan to promote resilience and support well-being	Increasing insight and motivation
2. Motivation and change	Triggering and maintaining mechanisms. Pros and cons of making changes. Setting a specific treatment goal	Fostering intrinsic motivation and hope
3. Balancing food and activity	The body’s energy needs, the role of physical activity, and the development of unbalanced patterns. Creating a food and activity plan. Introducing *My Day*, a daily self-monitoring tool for tracking emotions, eating patterns, and compensatory behaviours. Introducing *Exercise Cards*, a tool designed to support behavioural change	Modification of maladaptive thoughts and behaviours. Establishing habits
4. Involving others	Eating disorders impact on interpersonal relationships. The role of significant others in the recovery process. Openness and strategies for practicing transparency	Increasing insight and establishing habits
5. Management of emotions	The nature and function of emotions. Emotions impact on eating disorders. Emotion regulation and dysfunctional emotional behaviours. Introducing strategies for identifying, understanding and manging overwhelming emotions	Modification of maladaptive thoughts and emotions
6. Managing social situations	Eating disorders impact on social situations. Identifying maladaptive thoughts and behaviours. Introducing exposure training. Developing an exposure exercise/plan	Increasing insight and establishing habits
7. Improving self-esteem	Negative self-evaluation and eating disorders. The nature of self-esteem. Introducing strategies for practicing a healthier sense of self-worth	Modification of maladaptive thoughts
8. Preventing relapse	Reviewing progress, planning for relapse prevention, and preparing for continuing skill use. Developing a relapse prevention plan	Implementing durable changes in habits
Common features across modules	Psychoeducation. Normalisation. Personal formulations Behavioural experiments. Exposure exercises. Registration and planning. The use of personas: Semi-fictional and research-based persons representing the adolescents who will use the interventions	Increasing; insight, hope, motivation, relatedness, competence, autonomy, and generalisation of newly acquired skills

### Therapist guidance

The weekly therapist telephone guidance included assessment of adolescents’ progress, focusing on content and module tasks from the previous week. Adolescents were able to send asynchronous messages to therapists via the application. Messages related to treatment content were addressed during the scheduled telephone consultations, whereas messages concerning scheduling, technical, or practical issues were addressed on an ongoing basis. After completing the eight treatment modules, a follow-up video consultation was conducted to review treatment progress and evaluate the need for further care. The clinicians participating in this study, had a variety of professional backgrounds and training including one clinical psychologist, one social worker and three mental health nurses. All had prior experiences with the target population, but not with delivering digital treatments. Manualised instructions were developed to guide the clinicians. In addition, they received a brief introductory course on the functionality of the digital platform and on how to deliver the intervention. The clinicians were required to attend a one-hour group supervision sessions every fortnight, led by a senior clinical psychologist. The primary purpose of supervision was to give clinicians the opportunity to discuss the therapeutic content of the program. In addition, the sessions allowed them to address technical aspects of the digital platform and raise therapeutic concerns or questions, for example, whether a patient was too unwell to participate or how to proceed when motivation was low. Decisions regarding whether a participant required more intensive treatment than the digital intervention could provide were made by clinicians with overall clinical responsibility at each site. At Haukeland University Hospital, a child and adolescent psychiatrist held this responsibility, and at Helse Fonna it was a clinical psychologist specialist. The clinicians delivering the intervention monitored participants’ clinical status throughout the treatment and reported any concerns to the responsible clinicians, who then ensured that any need for higher-level care was appropriately identified and addressed. A member of the research team was participating in the sessions to ensure adherence to the research protocol.

### Measures

All measures were digitally delivered self-report questionnaires. A summary of measures and assessment timepoints are outlined in Table 3.

**Table 3:** Outcome measures and time of assessment

Outcome measures	Pre-treatment	Mid-treatment	Post-treatment
Participant characteristics Age Gender Living situation Engagement in school Engagement with friends History of present illness	x x x x x x	– – – – – –	– – – – – –
Acceptability Module evaluation (1-item) Acceptability questionnaire (15-item)	– –	x* –	– x
Adherence Module completion Treatment drop-out	– –	– –	x x
Primary clinical outcomes Eating disorder symptoms (EDE-QS) Psychosocial impairment (CIA)	x x	x x	x x
Secondary clinical outcomes Quality of life (KIDSCREEN10) Emotion dysregulation (DERS-18) Depression (PHQ-2) Anxiety (GAD-2)	x x x x	x x x x	x x x x

Mid-treatment: After five weeks

*: Assessed after each module

### Participant characteristics

Data regarding age, gender (girl/boy/other gender identities), living situation, engagement in school/with friends and history of present illness were gathered pre-treatment using a questionnaire developed specifically for this study.

### Treatment acceptability

Perceived acceptability of the digital treatment was assessed from feedback provided by the participants during and post treatment. After completing each module, participants were asked whether they experienced the module as useful. This was assessed using a 5-point Likert-type scale, where participants could choose between: Strongly disagree (1), disagree (2), neither disagree nor agree (3), agree (4), and strongly agree (5). At the post treatment assessment, a 15-items questionnaire previously used in adult populations [43] was adapted to be age-appropriate and suitable for the current study context. The questionnaire included multiple-choice and open-text questions. It assessed participants’ perceptions of the digital treatment, including treatment components and factors influencing completion. It also measured treatment credibility by asking whether participants would recommend the treatment to a friend with similar problems [44, 45]. The questionnaire was not intended to generate a single overall score; instead, each item was examined and reported separately.

### Treatment adherence

Adherence was evaluated by assessing engagement with the treatment modules and dropout. More specifically the average number of modules completed, and the number of dropouts is reported. In this study, treatment dropout was defined as participants who completed pre-treatment assessment and interacted with the eBalance program but completed less than six modules (< 75%). Pre-treatment dropout was defined as participants who consented to participate int the study but never enrolled in the intervention. In addition, reasons for non-adherence were addressed.

### Preliminary effectiveness

The interventions preliminary effectiveness was assessed by examining symptoms change from pre to post treatment. Treatment outcome measures were chosen based on the intervention’s aims.

#### Primary clinical outcomes

The following symptoms were measured as primary clinical outcomes:

##### Eating disorder symptomatology

The 12-item Eating Disorder Examination-Questionnaire Short (EDE-QS) was used to explore disordered eating attitudes and behaviours [46]. Total scores range from 0 to 36, where higher scores indicate greater severity of eating disorder symptomatology. Previous research [46], including studies in Norwegian samples [47], supports the EDE-QS as a valid and reliable measure. In the present study, internal consistency was good, with Cronbach’s alpha of 0.82 at the pre-treatment assessment.

##### Psychosocial impairment

Severity of psychosocial impairment due to eating disorder features was assessed using the 16-item questionnaire Clinical Impairment Assessment Questionnaire (CIA) [48]. In addition to a global scale, three sub-scales represent different areas of impairment that can result from eating disorders (personal impairment, social impartment and cognitive impartment). Global scores range from 0 to 48, where higher scores indicate greater severity of impairment. Previous research [48], including studies in Norwegian samples [49], supports the CIA as a valid and reliable measure. In the current study, the CIA global score showed excellent internal consistency at the pre-treatment assessment, with Cronbach’s alpha of 0.94. In addition, the Social Impairment subscale demonstrated excellent internal consistency with Cronbach’s alpha of 0.90, while the Personal Impairment and Cognitive Impairment subscales showed good internal consistency with Cronbach’s alpha values of 0.88 and 0.81, respectively.

#### Secondary clinical outcomes

The following symptoms were measured as secondary clinical outcomes:

##### Quality of life

The ten-item version of KIDSCREEN was used to assess health-related quality of life [50]. The KIDSCREEN-10 provides an index score from 10 to 50, where higher scores indicate better overall well-being, including aspects physical, mental, and social well-being. Previous research [51], including studies in Norwegian samples [50], supports the KIDSCREEN as a valid and reliable measure. In the current study, internal consistency at pre-treatment assessment was good, with Cronbach’s alpha of 0.80.

##### Emotion dysregulation

The 18-item version of the Difficulties with Emotion Regulation Scale (DERS-18) was used to assess level of difficulties with emotion regulation [52]. Scores range from 18 to 90, where higher scores indicate more severe emotional dysregulation. The DERS-18 has demonstrated good validity and reliability across clinical and non-clinical samples [53]. A Norwegian validation study is not jet published. In the current study, internal consistency for the total scale at pre-treatment assessment was excellent, with Cronbach’s alpha of 0.91.

##### Depression

The two-item version of the Patient Health Questionnaire (PHQ-2) was used to assess symptoms of depression [54]. Scores range from 0 to 6, where higher scores indicate more severe symptoms. The PHQ-2 is a widely used valid and reliable screening measure [55, 56]. In the present study, internal consistency at pre-treatment assessment was good, with Cronbach’s alpha of 0.83.

##### Anxiety

The two-item version of the Generalized Anxiety Disorder scale (GAD-2) was used to measure symptoms of anxiety [57]. Scores range from 0 to 6, where higher scores indicate more severe symptoms. The GAD-2 has demonstrated good validity as a brief screening measure for anxiety symptoms [56, 57]. In the current sample, internal consistency at pre-treatment assessment was good, with Cronbach’s alpha of 0.82.

### Statistical analyses

All analyses were conducted using SPSS Statistics version 29. Descriptive statistics were used to determine participant characteristics, acceptability measures and adherence. Linear mixed models for repeated measures analysis were performed to test clinical outcome data on the group level at pre-treatment, mid-treatment, and at post-treatment. Missing data were estimated from the observed data under a missing-at-random assumption, using a restricted maximum likelihood as recommended by Chakraborty and Gu [58]. This assumption implies that the probability of missing observations could be explained by variables already measured, such as baseline characteristics and earlier symptom scores, rather than by the unobserved values themselves. Effect sizes (Cohen’s d) were calculated for pre-mid and pre-post change based on differences between timepoints using the following formula by Morris and DeShons’: *d* = $\frac{{M}_{Post}- {M}_{Pre}}{{SD}_{Pre}}$ [59]. Standard deviation (SD) was calculated by$Standard Error \left(SE\right)\times \surd n$. Reliable change was assessed using the Reliable Change Index (RCI). RCI was selected due to its widespread use as a standard method for evaluating individual-level change in clinical research [60, 61]. As is common practice, the RCI was calculated by taking each individual change score (pre-post) and then dividing this score by the standard error of the difference using the formula: *RCI*= $\frac{{X}_{Post}- {X}_{Pre}}{{SE}_{diff}}$ [60]. Standard error of difference (SEdiff) was computed using the following formula: $SEdiff\, = \sqrt 2 (SEM)^{2}$ , while standard error of measurement (SEM) was computed using the formula: $SEM = SD \times \sqrt {(1 - r)}$. In line with recommendations by Lambert and Ogles (2009), we employed normative data for standard deviation and the measurements internal consistency estimate (Cronbach's alpha) for measuring reliability [62], given the limitations imposed by a small sample size. Participants who exhibited a change from pre to post treatment exceeding the reliable change boundaries (± 1.96) were classified as having achieved reliable improvement or deterioration, respectively. Change scores falling within these boundaries were classified as not reflecting reliable change. RCI was used to assess potential negative effects of the intervention by examining deterioration in primary outcomes.

## Results

### Recruitment and study flow

**Fig. 1:**
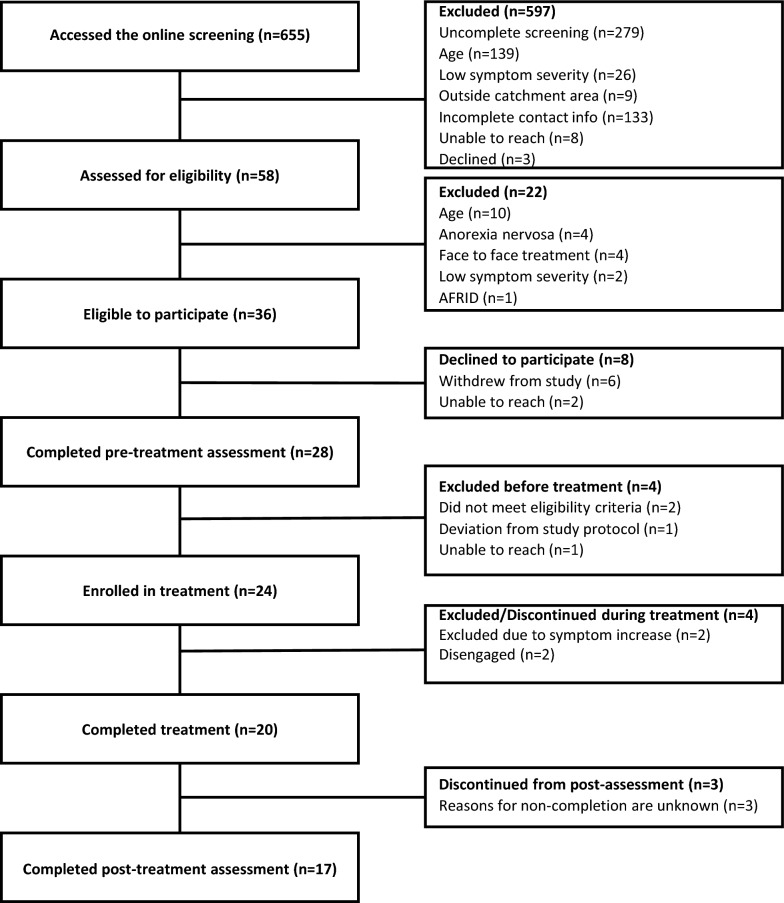
Recruitment flowchart

A total of 655 individuals accessed the online screening portal between March 2024 and May 2025 (Fig. 1). A total of 58 participants were assessed for eligibility through diagnostic telephone screening, of whom 36 were eligible to participate in the study. Participants were recruited through CAMHS (n = 20), upper secondary schools (n = 8), and social media (n = 8). A total of 28 participants provided informed consent and completed the pre-treatment assessment. Four were excluded prior to treatment enrolment due to meeting diagnostic criteria for anorexia nervosa (n = 1), bulimia nervosa (n = 1), deviation from study protocol (n = 1), and non-response to therapist contact (n = 1). Of the 24 participants enrolled, 20 completed treatment and 17 completed the post-treatment assessment.

### Participant characteristics

All the participants were girls (Table 4). The median age of the study sample was 16 years (SD = 0.78, range 15–18). Over half of the sample was living with both parents (58%), all were engaged in school (100%), and almost all (96%) were engaged with friends on a regular basis. At inclusion, the most frequent diagnosis was eating disorder, unspecified (33%), followed by atypical anorexia nervosa (29%), binge eating disorder (21%), and atypical bulimia nervosa (17%). Half of the adolescents had previously received face-to-face treatment for an eating disorder (50%). The mean age of eating disorder onset was 13 years (SD = 1.83, range 9–16).

**Table 4:** Participants characteristics (N = 24)

Variable	Values, n (%)
Gender Boy Girl Other gender identities	0 (0) 24 (100) 0 (0)
Mean age	16.46 (SD = 0.78)
Living situation Both parents 50/50 residential One parent Alone Other living agreement	14 (58) 3 (13) 3 (13) 2 (8) 2 (8)
Engaged in education (total) Reduced time Full-time	24 (100) 5 (21) 19 (79)
Engaged with friends (total) Daily Weekly Monthly	23 (96) 6 (25) 10 (42) 7 (29)
Diagnoses (inclusion) Atypical anorexia nervosa Atypical bulimia nervosa Binge eating disorder Eating disorder, unspecified	7 (29) 4 (17) 5 (21) 8 (33)
Prior eating disorder treatment Yes No	12 (50) 12 (50)
Mean age of disorder onset	12.71 (SD = 1.83)

### Treatment acceptability

#### Module evaluation

Table 5 summarises the adolescents’ experiences regarding the usefulness of the eight modules. All modules were perceived as useful, with agreement rates ranging from 70% for Module 5 (17/24) to 100% for Module 7 (13/24). Median item response scores were four (agree) across all modules.

**Table 5:** Participants rating of the modules usefulness

I found this module useful	N	M	n (%*)	n (%*)	n (%*)	n (%*)	n (%*)
			Strongly agree	Agree	Neither disagree nor agree	Disagree	Strongly disagree
1. What is an eating disorder?	24	4	1 (4)	16 (67)	4 (17)	3 (13)	–
2. Motivation and change	24	4	5 (21)	15 (63)	4 (17)	–	–
3. Balancing food and activity	22	4	4 (18)	15 (68)	2 (9)	1 (5)	–
4. Involving others	18	4	3 (17)	11 (61)	4 (22)	–	–
5. Management of emotions	17	4	5 (29)	7 (41)	5 (29)	–	–
6. Managing social situations	14	4	3 (21)	8 (57)	3 (21)	–	–
7. Improving self-esteem	13	4	4 (31)	9 (69)	–	–	–
8. Preventing relapse	13	4	3 (23)	9 (69)	1 (8)	–	–

*N* Number of participants answering the item, *M*: Median

*: Valid percent

#### Acceptability questionnaire

The results from the acceptability questionnaire demonstrated that the treatment was perceived as acceptable for most of the adolescents who completed the questionnaire (n = 15). A detailed description of the responses is presented in Additional file 2. Overall, 12 out of the 15 adolescents (80%) considered the treatment credible and appropriate for their problems. A total of 13 adolescents (87%) reported being satisfied with the treatment and felt it brought about positive changes. Open-ended responses to the positive outcomes of the treatment included (n = 15): *“Less compensation,” “More social, less exercise and not counting calories”, “I feel like things have finally gotten better”, “I manage to eat regularly, "I learned a lot about myself and the things I struggle with”, "I often eat a bit too much, but I don’t think about food all the time anymore. I feel a bit more free and more motivated to continue losing weight”, "That I will learn to accept myself just the way I am”,” “New ways of thinking,”* and *“Felt less alone”.* Eight adolescents (53%) reported no negative changes from the treatment, four (27%) indicated small to some negative changes, and three (20%) reported quite a lot or a lot of negative changes. Open-ended responses to the negative outcomes included (n = 14): “*Too much focus on the problem”, “It would have been better if it hadn't been digital because I felt alone”, “I felt I was not sick enough to be taken seriously”, “I don’t know”, “An abrupt ending. I’ve been in treatment for the past three years, so when I suddenly had to manage everything on my own, it was overwhelming”, “The reading”,* “*It took quite a long time before I managed to change my habits, but things are getting better the past month. I’ve also not prioritised the treatment, been very busy or just forgotten it”,* and *“It wasn’t the treatment itself that made me feel worse. The only issue was the commitment I made and the pressure to complete it, even when I neither wanted to, nor had the strength to do so”.*

Moreover, 10 of the 15 adolescents (67%) reported they would have benefited less from the treatment without therapist-guidance, while five (33%) felt they would have benefited equally. Open-ended responses to what participants felt was missing in their contact with the therapist included (n = 14): *“Did not miss anything, but I prefer face-to-face meetings”, “It felt a bit impersonal at times, when I only heard a voice. Maybe it would have been better on video or face-to-face”,* and* “It was difficult to talk on the phone, I was just sitting there waiting for it to be finished”.*

#### Treatment adherence

Of the 24 adolescents enrolled in treatment, participants completed an average of seven out of eight modules (88%), while the median completion was eight modules. Using this study’s definition of treatment dropout (completing less than six modules, < 75%), two adolescents (8%) discontinued prematurely, and two (8%) were excluded during treatment. The adolescents’ reasons for discontinuing treatment were: i) perceived improvement and reduced need for support, making the treatment feel too time-consuming (disengaged after Module 3; completing 38% of the treatment) and ii) a somatic condition resulting in low energy and limiting engagement with the treatment (disengaged after Module 5; completing 63% of the treatment). Clinicians excluded two adolescents during treatment for the following reasons: (i) increased vomiting frequency meeting diagnostic criteria for bulimia nervosa (excluded after Module 3; completed 38% of treatment) and (ii) worsening symptoms of a comorbid condition (PTSD) (excluded after Module 3; completed 38% of treatment).

#### Treatment outcomes

Results of the linear mixed model analyses for the primary and secondary clinical outcome measures are summarised in Table 6.

**Table 6:** Overview of results from the linear mixed model analyses of change over time across primary and secondary outcomes (n = 24)

	Pre treatment		Mid treatment		Post treatment		Pre to mid					Pre to post				
	M (SD)		M (SD)		M (SD)		b	SE	df	p	d	*b*	*SE*	*df*	*p*	*d*
EDE-QS	21.96	(7.35)	19.28	(7.54)	18.62	(7.74)	− 2.68	0.92	36.38	0.006*	− 0.36	− 3.34	0.98	36.55	0.002*	− 0.45
CIA global score	27.92	(11.32)	28.17	(11.66)	25.02	(12.00)	0.25	1.54	36.32	0.871	0.02	− 2.90	1.64	36.52	0.085	− 0.26
CIA personal impairment	13.08	(4.36)	12.70	(4.56)	13.14	(4.70)	− 0.38	0.68	36.27	0.575	− 0.09	0.05	0.72	36.55	0.942	0.01
CIA social impairment	7.79	(3.87)	7.42	(4.02)	6.09	(4.16)	− 0.38	0.59	36.55	0.525	− 0.10	− 1.70	0.62	36.81	0.010*	− 0.44
CIA cognitive impairment	7.04	(4.02)	8.08	(4.12)	5.84	(4.26)	1.04	0.58	36.49	0.079	0.26	− 1.20	0.61	36.72	0.057	− 0.30
KIDSCREEN	28.46	(5.49)	27.87	(5.88)	29.52	(6.12)	− 0.59	0.98	35.26	0.553	− 0.11	1.07	1.04	35.60	0.314	0.19
DERS	55.00	(13.33)	54.81	(13.91)	52.47	(14.35)	− 0.19	1.96	34.07	0.925	− 0.01	− 2.53	2.09	34.27	0.235	− 0.19
PHQ	2.83	(1.67)	2.92	(1.81)	2.65	(1.91)	0.08	0.32	35.63	0.795	0.05	− 0.18	0.34	36.05	0.591	− 0.11
GAD	3.54	(1.71)	3.63	(1.81)	3.26	(1.96)	0.08	0.34	33.60	0.807	0.05	− 0.29	0.37	34.26	0.445	− 0.16

*EDE-QS* Eating Disorder Examination-Questionnaire Short, *CIA* Clinical Impairment Assessment Questionnaire, *KIDSCREEN* Health-Related Quality of Life Questionnaire (reversed score), *DERS* Difficulties with Emotion Regulation Scale, *PHQ* Patient Health Questionnaire, *GAD* Generalized Anxiety Disorder scale, *M* mean, *SD* Standard Deviation, *b* regression coefficient (beta), *SE* Standard Error, *df* degrees of freedom

*Significance value

d: Cohen's d calculated from differences between time points

#### Primary clinical outcomes

Primary clinical outcomes are presented in Table 6. A statistically significant improvement in eating disorder symptomatology (EDE-QS) was observed from pre to mid treatment (*p* = 0.006, *d* = 0.36) and from pre to post (*p* = 0.002, *d* = 0.45). Psychosocial impairment (CIA global score) had no significant change from pre to mid (*p* = 0.871, *d* = -0.02) or from pre to post (*p* = 0.085, *d* = 0.26). However, a statistically significant improvement was observed from pre to post treatment (*p* = 0.010,* d* = 0.44) on social impairment (CIA social subscale). No significant changes were observed on the CIA subscales cognitive impairment and personal impartment.

#### Secondary clinical outcomes

None statistically significant improvements were observed in the secondary clinical outcome measures (Table 6).

### Reliable change analyses

Of the 17 participants who completed post-treatment assessment, a total of 11 participants (65%) showed reliable improvement in their EDEQS scores, whereas 4 participants (24%) remained unchanged and 2 (12%) deteriorated. Reliable change on the CIA scores showed that 7 (41%) showed reliable improvement, 9 (53%) remained unchanged and 1 (6%) participant deteriorated. Two participants (12%) experienced deterioration in primary outcomes. One participant experienced deterioration in both EDE-QS and CIA scores, while one in EDEQS.

## Discussion

To our knowledge this is the first study to evaluate a therapist-guided digital treatment for adolescents with subthreshold eating disorders within routine clinical care. The findings indicate the treatment is feasible, with favourable ratings of acceptability and satisfactory adherence rates. Preliminary effectiveness was promising, with a significant improvement in eating disorder symptomatology and social impairment. Non-significant improvements were observed for quality of life, emotion dysregulation, anxiety, and depression.

## Recruitment

Although a substantial number of individuals accessed the online screening portal, it took over a year to recruit 28 adolescents to participate in the digital treatment. Recruitment challenges are common in clinical research [63]. In this study it could be due to several reasons for example the ambivalent nature of eating disorders [64] or the participants being adolescents [65]. The fact that as many as 141 individuals met the criteria for the online screening but did not leave their contact nor respond to the phone call, might reflect well-documented barriers to help-seeking behaviours in eating disorders [66]. Other reasons for recruitment challenges could be doubt regarding digital interventions effectiveness [21] or that individuals with eating disorders may still prefer face-to-face treatment [67]. Future studies will aim to include adolescents with anorexia nervosa and bulimia nervosa, which is expected to increase the number of eligible participants.

## Treatment acceptability and adherence

The digital treatment was perceived as useful, credible, and satisfactory, indicating high acceptability. Acceptability of digital interventions for eating disorders has been explored in only a limited number of studies, with findings showing considerable variation [19]. In addition, this study demonstrated satisfactory adherence rates [68]. This contrasts with other trials where adherence to digital interventions is suboptimal [17, 21, 69]. There are several potential explanations for the good acceptability ratings and the good treatment adherence observed in this study. First, this can be attributed to the intervention being designed and developed based on the perspectives of its intended users [39, 40, 69, 70]. Many interventions tested in young people have originally been developed for adult populations, rather than being specifically designed and co-designed with adolescents themselves [20, 71]. Interventions that fail to consider users’ needs and preferences are known to contribute to low adherence [21, 69]. A second reason can be attributed to the intervention being therapist guided. Studies show that therapist support increases satisfaction with intervention [19] and adherence among adolescents is relatively weak if not boosted by in-person elements [72]. Adherence to digital treatments may improve when adolescents feel accountable to a respected and credible support person [21]. This aligns with studies showing that dropout rates are poorer in self-guided digital interventions [69, 73]. In addition, the result from this study can be attributed to the digital treatment being delivered via a smartphone application. This enables the adolescents to approach treatment at their own pace and could help them feel more in control of their treatment [20]. Providing the adolescents with a sense of control and agency in treatment decision-making are factors known to enhance adherence to therapeutic interventions [20, 21].

## Preliminary effectiveness

By examining symptom change across the duration of treatment, the intervention’s preliminary effectiveness on eating disorder symptomatology was supported. The analyses demonstrated clinically significant improvement in eating disorder symptomatology with a medium effect size. The Reliable Change Index confirmed that 11 of the 17 adolescents (65%) who completed the post-assessment showed a reliable improvement in eating disorder symptomatology, exceeding what could be attributed to random measurement error [60]. Notably, significant improvements were already observed at the mid-treatment assessment, likely due to the completion of Modules 1 to 3, which primarily target disordered eating attitudes and behaviours. Despite the significant improvements in eating disorder symptoms, the sample’s mean posttreatment score remained above the clinical cut-off of > 15 (EDE-QS = 18.62). A recent study examining the questionnaire’s psychometric properties among Norwegian adolescents, however, suggests that a total score of 20 may represent a more appropriate clinical cut-off [47]. Participants completed follow-up assessments at 3 and 6 months, enabling evaluation of whether the reliable improvements in eating disorder symptomatology are sustained beyond the initial treatment phase. These findings will be presented in a forthcoming paper. Longitudinal data are particularly important in app-based interventions, as both engagement patterns and treatment effects may fluctuate after the active treatment period has ended [26, 74]. For the study’s other primary clinical outcome, a significant effect with a medium effect size was observed in the social impairment subscale. Based on input from adolescents with lived experience of eating disorders, the treatment included a module focusing primarily on managing social situations (Module 6) [39]. This may serve as a potential explanation for the decrease in social impairment among participants. No clinically significant improvement was observed in the global psychosocial impairment score and the sample’s mean posttreatment score remained above the clinical cut-off of > 16. Moreover, only 7 out of 17 participants (41%) showed reliable improvement. The follow-up assessments will be essential for determining whether the severity of psychosocial impairment due to eating disorder features continues to improve over time. Moreover, a larger sample would have provided more precise estimates of treatment effects and enabled the identification of subgroups of potential responders.

Although the intervention included a module on emotion management (Module 5), based on input from adolescents with lived experience [39, 41] no significant effects were observed in this domain. Given the adolescents’ symptom severity and the fact that the module was perceived as useful, these findings underscore the importance of addressing emotion dysregulation in interventions for eating disorders. Furthermore, no significant effects were found on measures of anxiety and depression. Although previous trials have shown that improvements in eating disorder symptoms were sometimes accompanied by a deterioration in anxiety and depressive symptoms [75] the current sample did not experience such deterioration. The results from this study, combined with the fact that anxiety and depression are the most prevalent comorbid disorders in adolescents [2], underscore the importance of further evaluating whether Module 5 (management of emotions) should be redesigned and expanded to incorporate more content targeting these domains.

Overall, there is a limited number of studies investigating digital treatment for eating disorders in adolescents [16, 19, 75]. Especially research on app-based interventions for eating disorders has been relatively limited [20, 24]. Findings from studies in older cohorts may not be directly comparable, as older individuals might be less familiar or comfortable with newer technologies [19]. While earlier studies in adolescents tested applications as adjunction to traditional therapy, our study examined a stand-alone intervention. Despite the absence of concurrent face-to-face treatment, the promising results from the current study suggest that a fully app-delivered approach may hold independent therapeutic value. Taken together, the preliminary effectiveness of the current digital treatment give reason for cautious optimism but given the design and nature of the current study, it is important to examine the treatment further in a randomized controlled trial with a larger sample size to determine any clinical effects.

## Limitations

This study has several limitations that needs to be addressed. Firstly, due to small sample size and the lack of control group the results from this study should be taken as preliminary. Secondly, due to lack of appropriate norm data for the assessed population, the study used data from a community-based sample when calculating RCI for EDE-QS [76] and for adults when calculating RCI for CIA [49]. Thirdly, only 17 of the 24 adolescents enrolled in treatment completed the post-assessment. Although we estimated the missing data, there is inherent uncertainty in these estimates. For instance, in the acceptability assessments, data were available only from participants who completed certain items or the entire questionnaire. It remains unclear whether this pattern of missingness reflects that those who were more satisfied with the treatment were more likely to respond. Moreover, a limitation is that module-level engagement data (e.g., time spent per module and time to completion) was not included in the present study, which prevented a more detailed analysis of participants’ interaction with the intervention. Furthermore, the participants do not fully represent the broader population of adolescents with eating disorders, as the sample lacked gender diversity and excluded those with anorexia nervosa and bulimia nervosa. In addition, the study did not assess the acceptability among the clinicians. This limits the results regarding the feasibility within routine clinical care and might hinder further implementation of the intervention. Lastly, the qualitative data in this study are limited by the fact that they were derived solely from open-ended questionnaire responses rather than in-depth interviews, which would have provided a richer and more nuanced understanding of participants’ experiences with the treatment.

## Clinical implications and future perspectives

Although this study has certain limitations, it also offers several clinical implications that warrant consideration. The digital treatments perceived usefulness, satisfaction and credibility are promising in terms of relevance of the intervention to complement or extend traditional eating disorder care for adolescents. The digital intervention was originally developed drawing on qualitative insights from adolescents with lived experience of anorexia nervosa, bulimia nervosa, and binge eating disorder [39]. The present study focused on adolescents with subthreshold eating disorders; however future research should examine the intervention’s acceptability and effectiveness across a broader spectrum of eating disorder diagnoses. While Other Specified Eating Disorder (a subthreshold disorder) represent the most prevalent eating disorder among adolescents, it is followed in frequency by bulimia nervosa and anorexia nervosa [77]. Given the large number of adolescents affected by these disorders [77] and the many who remain untreated [9, 66], it is important to assess whether the intervention provides comparable benefits across diagnostic groups and to determine whether content adaptations are required to ensure it can effectively contribute to reducing the current gap in care.

The adolescents’ engagement with treatment content and low dropout rate are important considering there is some evidence that greater adherence is linked with greater reductions in mental health problems [69]. However, despite high acceptability and adherence, the result from this study underline that this treatment format may not have been suitable for all participants. Some adolescents reported negative changes from the treatment, and a proportion did not show reliable improvement in primary or secondary clinical outcomes. In addition, some of the adolescents’ open-ended responses indicated that the digital format and telephone conversations felt less personal, and that they would have preferred face-to-face or video-based contact. A larger sample would allow for comparisons of adolescents’ characteristics, responses, and outcomes, and help identify potential predictors of who might benefit most from a digital treatment.

The fact that treatment was delivered within routine clinical care highlights the intervention’s acceptability, adherence, and preliminary effectiveness in a real-world setting. The results from this study will help determine whether routine clinical care could be a suitable context for delivering a therapist-guided digital treatment for adolescents with eating disorders. Trials often bear little resemblance to clinical settings which make further implementation and sustainment difficult [63]. However, a more rigorous study is needed, not only for this treatment, but in general regarding digital interventions for eating disorders [10, 19]. In addition to a control group, follow-up assessments need to the included to evaluate the long-term effectiveness of the intervention. Moreover, the findings from this study underscore the well-established need to include diverse populations in future research. Lastly, conducting the intervention in line with the UK Medical Research Council’s guidance for developing and evaluating complex interventions strengthens the study’s clinical relevance. This framework ensures a systematic, theory-driven, and user-informed development process, increasing the likelihood that the intervention is feasible [29]. However, the absence of clinician data in this study limits the assessment of its relevance for routine clinical care. Clinicians will be included in future evaluations in accordance with MRC guidance, and these findings should be reported to inform potential barriers and facilitators to implementation for other developers.

## Conclusion

Findings showed that the adolescents were overall satisfied with the therapist-guided digital intervention and perceived the treatment to be helpful. The result from this study suggest that a therapist-guided digital treatment could be well tolerated and accepted among adolescents with subthreshold eating disorders within routine clinical care. These findings highlight the potential of digital interventions to complement existing treatment pathways effectively.

## Supplementary Information

Additional file 1: CONSORT 2010 checklist of information to include when reporting a pilot or feasibility trial. Reporting guidance for feasibility trial.

Additional file 2: Acceptability questionnaire. A detailed description of the responses.

## Data Availability

Data generated, analysed, and reported during the current study are not publicly available, but are available in a slightly shortened, de-identified form from the corresponding author on reasonable request.
