# Supplementary material for: The acceptability, adherence, and preliminary effectiveness of a digital treatment for adolescents with subthreshold eating disorders. Findings from an open feasibility trial in routine clinical care
Source: J Eat Disord. 2026 Apr 9;14:115. doi: 10.1186/s40337-026-01596-9 (PMC13182040; doi:10.1186/s40337-026-01596-9)
Supplement: Supplementary file 2 — Additional file 2: Acceptability questionnaire. A detailed description of the responses. [file 40337_2026_1596_MOESM2_ESM.pdf]

Acceptability questionnaire

	Items	N	Median	n (%)	n (%)	n (%)	n (%)	n (%)	n (%)
1	How satisfied are you with the treatment?	15	5	Not satisfied at all<br>1 (8)	Mostly dissatisfied<br>1 (8)	Somewhat satisfied<br>-	Mostly satisfied<br>4 (27)	Satisfied<br>7 (47)	Very satisfied<br>2 (14)
2	To what extent do you feel things have changed in a positive direction?	15	3	Not at all<br>2 (13)	Small extent<br>3 (20)	Some extent<br>6 (40)	Quiet a lot<br>2 (13)	A lot<br>1 (7)	Very much<br>1 (7)
4	To what extent do you feel things have changed in a negative direction?	15	1	Not at all<br>8 (53)	Small extent<br>3 (20)	Some extent<br>1 (7)	Quit a lot<br>2 (13)	A lot<br>1 (7)	Very much<br>-
6	How appropriate do you feel the treatment was for your problems?	15	5	Completely inappropriate<br>1 (7)	Mostly inappropriate<br>1 (7)	Somewhat inappropriate<br>1 (7)	Somewhat appropriate<br>3 (20)	Mostly appropriate<br>8 (53)	Completely appropriate<br>1 (7)
7	How demanding did you find the treatment?	15	4	Far too demanding<br>1 (7)	Very demanding<br>2 (13)	Demanding<br>4 (27)	Somewhat demanding<br>7 (47)	Not very demanding<br>1 (7)	Minimally demanding<br>-
8	To what extent did you feel you were able to meet the demands of the treatment?	15	3	Not at all<br>1 (7)	Small extent<br>2 (13)	Some extent<br>5 (33)	Fairly well<br>7 (47)	Without difficulty<br>-	Could be more demanding<br>-
9	Did you gain more knowledge about eating disorders?	14	1	No<br>1 (7)	Yes<br>9 (60)	Unsure<br>4 (27)	-	-	-
10	If a friend needed similar help, would you recommend this treatment?	15	3	Definitely not<br>1 (7)	Probably not<br>2 (13)	Probably yes<br>8 (53)	Definitely yes<br>4 (27)	-	
11	How did the technical aspects work?	15	4	Very poorly<br>-	Poorly<br>1 (7)	Neither good nor poorly<br>-	Good<br>10 (67)	Very good<br>4 (27)	-
12	Was there too much or too little text?	15	2	Too little<br>-	Just right<br>9 (60)	Too much<br>2 (13)	Good<br>3 (20)	Very good<br>1 (7)	-
13	How actively did you engage with the self-reflections/ exercises?	15	2	Did none<br>-	Did a few<br>10 (67)	Worked frequently<br>4 (27)	Worked often<br>1 (7)	Completed all<br>-	-
14	Would you have benefited equally from the treatment without the weekly therapist sessions?	15	3	Done better<br>-	Done equally<br>5 (33)	Done slightly worse<br>7 (47)	Done much worse<br>3 (20)	-	-

Note. Items 3, 5 and 15 had open-ended response and are summarised in text.
